# Decomposition and Comparative Analysis of the Prevalence of and Factors Associated With Smoking Between the Rural and Urban Elderly Population in China: A National Cross-Sectional Study

**DOI:** 10.3389/fpubh.2022.824587

**Published:** 2022-03-17

**Authors:** Lei Yuan, Zhe Zhao, Jin Wang, Maolin Du, Yan Xiao, Lijuan Liu, Jinhai Sun

**Affiliations:** ^1^Department of Health Management, Second Military Medical University, Shanghai, China; ^2^Department of Obstetrics and Gynecology, Beijing Aerospace General Hospital, Beijing, China; ^3^Department of Office, Second Military Medical University, Shanghai, China; ^4^Department of Medical and Research, Naval Medical Center, Second Military Medical University, Shanghai, China

**Keywords:** elderly, tobacco, smoking, rural, urban, China, cross-sectional study

## Abstract

This study aimed to compare and analyse the differences in smoking prevalence, and knowledge, attitudes, and factors associated with smoking between the rural and urban elderly population in China. In total, 6,966 participants aged 60 and above were included in this study, which assessed their smoking-related knowledge, attitudes, and perceptions toward tobacco control. The Chi-square test and logistic regression model were used for statistical analysis, and the Fairlie model was used for decomposition analysis. The overall prevalence of smoking was 25.6%; the rate was much higher in men than in women (overall: OR = 26.234; urban: OR = 31.260; rural: OR = 23.889). The rate of correct responses to all questions on smoking problems was significantly higher among the urban elderly than the rural elderly. Further, 64.18% of the participants supported printing photos of the health hazards of smoking on the cover of cigarette packs, and the rural elderly were more supportive of this. Moreover, only 36.52% of the participants supported increasing taxation and retail price of cigarettes; the urban elderly showed more support for this. Rules about smoking at home also played an important role, especially for families where smoking was not allowed at home, but with exceptions to the rule; however, this factor was only meaningful in urban families (urban: OR = 0.117). Through the Fairlie decomposition analysis, gender (-1.62%), age (-2.03%), region (13.68%), knowing about e-cigarettes (5.17%), rules about smoking at home (3.95%), and smoking-related knowledge scores (42.85%) were found to be associated with rural-urban disparities. This study focused on the differences in smoking between urban and rural areas in China. Smoking among the urban elderly was significantly less prevalent compared with the rural population. Factors including education, region, and smoking-related knowledge need to be addressed to reduce the gap between urban and rural health hazards in China.

## Introduction

With the rapid economic growth brought about by China's reform and opening-up policy, the living conditions of Chinese residents have improved rapidly, and education levels and health literacy have increased, leading to changes in resident's behaviors, increase in national exercise time, focus on balanced diet, and other factors; these changes have led to a continuous increase in life expectancy, as people have begun to pay greater attention to individual health (1–5). Interestingly, however, there is no significant change in tobacco consumption behavior (6, 7); according to the Chinese Center for Disease Control and Prevention (CCDC) survey report in 2019, although 81.8% of the people think that smoking is harmful to health, the smoking rate in China has not changed significantly (8). Current research evidence shows that smoking is a risk factor for several chronic diseases (6, 9, 10), and as a major tobacco producer and consumer of tobacco in the global market, China lies at the heart of this health crisis (11). According to a large-scale survey conducted by the CCDC in 2018, the prevalence of smoking among individuals aged 15 and above in China was 26.6%, slightly lower than 28.3% in 2010. In 2019, the prevalence of smoking in rural areas was 28.9%, and that in urban areas was 25.1%, indicating that a large number of people still continue to smoke (8).

Many medical studies have shown that smoking is more harmful to the health of the elderly (12–15). China's population is aging rapidly; according to the annual data of China's National Bureau of Statistics (16), 253 million people were over the age of 60 in 2019, accounting for 18.1% of the total population, an increase of 51.5% from 167 million in 2009. The rapid change in the population structure has further increased the disease burden of the elderly population in China. According to a survey conducted by the CCDC in 2019, 75.8% of people over 60 years of age in China suffered from one or more chronic diseases, and the presence of multiple diseases was relatively serious, especially for cardiovascular and cerebrovascular diseases, cancer, diabetes, and chronic respiratory diseases (17). The prevalence of hypertension, diabetes, and dyslipidemia was 58.3, 19.4, and 37.2%, respectively. According to the calculation of quality-adjusted life years, residents aged ≥ 70 years accounted for 39.1% of patients with central cerebrovascular diseases, 15.4% of those with cancer, 10.5% of COPD, 6.4% of Alzheimer's disease, and 2.2% of diabetes. The prevalence rate of chronic diseases in the urban elderly (79.6%) was higher than that in the rural elderly (73.8%); the prevalence rate in the eastern (78.0%) and central (77.1%) areas was significantly higher than that in the western (72.9%) areas (17).

In China, the health of the elderly is seriously threatened by tobacco, but there are many elderly smokers in both urban and rural areas (18). The goal of the Chinese government is to control the smoking rate among people aged 15 and above to 20% by 2030 (19). To achieve this goal, the elderly will be the focus of attention and the target group of policy implementation. This article is a special study on the smoking status of the elderly in China, and it is also the first large-scale investigation report on the smoking status of the elderly in China. We analyzed the smoking-related knowledge, attitudes, and behaviors of the elderly in China, and conducted a comparative analysis of the differences in smoking-related situations between the urban and rural elderly. We also evaluated the main factors associated with smoking among the elderly, aiming to provide reference data and suggestions for the implementation of tobacco control strategies in China and other countries.

## Materials and Methods

### Data Sources

**Figure 1:**
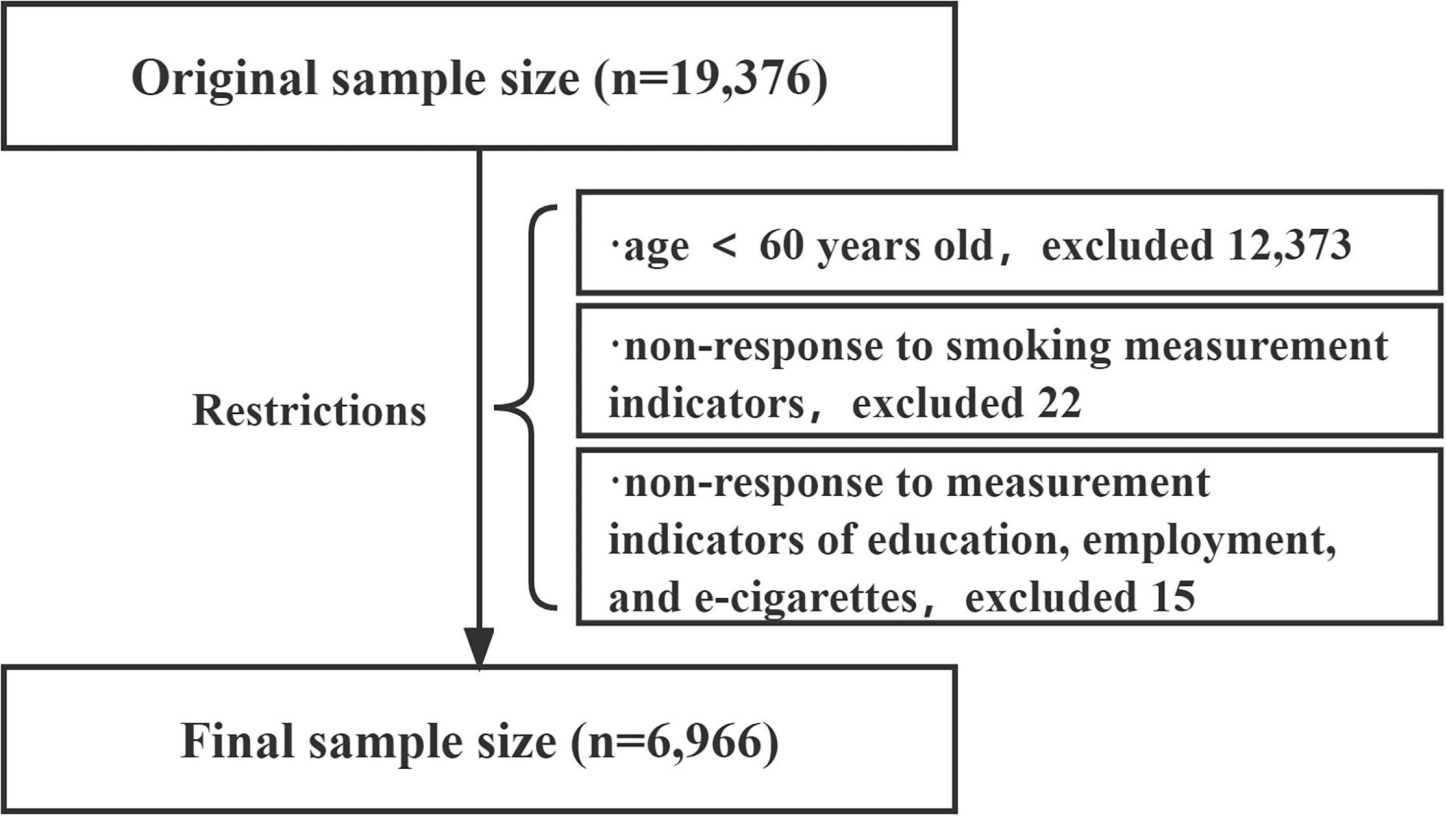
Flowchart of study participant.

The research data were taken from the Global Adult Tobacco Survey (GATS)-China 2018 (20); therefore, no ethics approval was required. The GATS China 2018 was a survey conducted by the CCDC. A total of 19,376 people were interviewed in 31 provinces in mainland China, using a multistage, stratified, cluster-randomized sampling design, recruiting community-based Chinese adults aged 15 years and older, Data available here (http://ghdx.healthdata.org/record/china-global-adult-tobacco-survey-2018, Accessed 26 Jan 2022). In each sampled geographic location, the households were randomly selected, and all eligible persons in each selected household were interviewed. However, only one household member was randomly selected and interviewed using a handheld device for rostering and data collection. Interviews were conducted privately by either male or female interviewers, the detailed design report is available here: (https://nccd.cdc.gov/GTSSDataSurveyResources/Ancillary/Publications.aspx, Accessed 26 Jan 2022). Based on our research needs, we selected 7,003 samples. After eliminating invalid data, 6,966 people were included in the study, the data processing flow is shown in Figure 1; there were 3,507 elderly people from urban areas and 3,459 from rural areas.

### Analytical Index

#### Demographic Data

Details regarding demographic factors included residence (urban or rural), gender (male or female), age, residential status (living alone or not alone), region (east, west, or central China), educational level (elementary education or below, secondary education, higher education, and above), annual income (<10,000 RMB, 10,000–29,999 RMB, 30,000–49,999 RMB, or ≥50,000 RMB), employment status (retired, working, or not working), current smoking status (yes or no), awareness of e-cigarettes (yes or no), and rules about smoking at home (allow, not allowed but with exceptions, never allowed, no rules, do not know, or refuse to answer).

#### Knowledge of Smoking

All participants were asked to respond to 10 items to evaluate smoking knowledge; the questions including statements on smoking and the relationship between smoking and the consequences of specific diseases, with 1 point for correct answers and 0 points for wrong or uncertain answers.

#### Attitudes Toward Smoking

Attitudes toward smoking were evaluated based on cigarette packaging and tobacco taxation. Respondents were shown pictures of cigarette packs with different health reminders to investigate intention of quitting among the elderly, and evaluate the role of cigarette packaging in tobacco control. The attitudes of the elderly toward tobacco taxation were evaluated through three questions and the differences in attitudes between the rural and urban elderly were analyzed.

### Statistical Method

Statistical analysis was performed using SPSS 21.0. Descriptive statistics were used to describe demographic data and the proportion of answers in different groups of questionnaires. Chi-square test was used to analyse the univariate of urban and rural, the test standard was α = 0.05, and multiple logistic regression model was established to evaluate the impact of different variables on smoking and quitting status (including α = 0.05, excluding α = 0.10). The Fairlie model was used for decomposition analysis; the construction of this model is explained in detail in Dr. Zhang's article (20). A two-tailed test of significance was conducted, with *P* < 0.05 as statistically significant.

## Results

### Demographic Characteristics

A total of 6,996 elderly individuals were included in this study, of whom 3,393 (48.71%) were men and 3,573 (51.29%) were women (Table 1). The mean age was 69.22 (SD = 7.251) years; 50.34% of the participants lived in urban areas, 25.27% were living alone, 68.42% had a low educational level, 58.63% had an annual income <30,000 RMB, and 58.73% still went to work. This survey showed that the prevalence of smoking among the elderly was 25.6%; the rate in urban areas was 23.0% and in rural was 28.3%, indicating a higher prevalence of smoking among the elderly people in rural areas (*P* < 0.001). Furthermore, more urban elderly people had heard of e-cigarettes (*P* < 0.001), and rural households were more likely to smoke (*P* < 0.001).

**Table 1:** Comparison of demographic characteristics and smoking status between the urban and rural elderly in China.

**Characteristics**	**Urban**	**Rural**	**χ^2^**	***P*-value**
	**[*n* (%)]**	**[*n* (%)]**		
Gender			7.257	0.007
Male	1,652 (47.1)	1,741 (50.3)		
Female	1,855 (52.9)	1,718 (49.7)		
Age			9.593	0.008
60–69 years	2,128 (60.7)	2,032 (58.7)		
70–79 years	960 (27.4)	1,057 (30.6)		
80 years or above	419 (11.9)	370 (10.7)		
Residential status			8.244	0.004
Living alone	834 (23.8)	926 (26.8)		
Not living alone	2,673 (76.2)	2,533 (73.2)		
Region			86.368	<0.001
East	1,592 (45.4)	1,196 (40.0)		
Central	1,021 (29.1)	1,249 (32.6)		
West	894 (25.5)	1,014 (29.3)		
Educational level			692.071	<0.001
Elementary education or below	1,919 (54.7)	2,847 (82.3)		
Secondary education	1,305 (37.2)	601 (17.4)		
Higher education and above	283 (8.1)	11 (0.3)		
Annual income			1031.529	<0.001
Very low (<10,000 RMB)	637 (18.2)	1,630 (47.1)		
Low (10,000–29,999 RMB)	822 (23.4)	995 (28.8)		
Middle (30,000–49,999 RMB)	822 (23.4)	426 (12.3)		
High (≥50,000 RMB)	1,084 (30.9)	295 (8.5)		
Don't know/Refuse	124 (4.0)	113 (3.3)		
Employment status			1959.293	<0.001
Retired	1,828 (52.1)	166 (4.8)		
Working	1,286 (36.7)	2,805 (81.1)		
Not working	393 (11.2)	488 (14.1)		
Current smoking status			26.152	<0.001
Smoking	805 (23.0)	979 (28.3)		
Not smoking	2,702 (77.0)	2,480 (71.7)		
Have you ever heard of e-cigarettes?			324.181	<0.001
Yes	1,010 (28.8)	397 (11.5)		
No	2,497 (71.2)	3,062 (88.5)		
Rules about smoking at home			424.361	<0.001
Allowed	995 (28.4)	1,387 (34.2)		
Not allowed but with exceptions	785 (22.4)	450 (13.0)		
Never allowed	1,131 (32.2)	607 (17.5)		
No rules	532 (15.2)	930 (26.9)		
Don't know/Refuse	64 (1.8)	85(2.5)		

**Figure 2:**
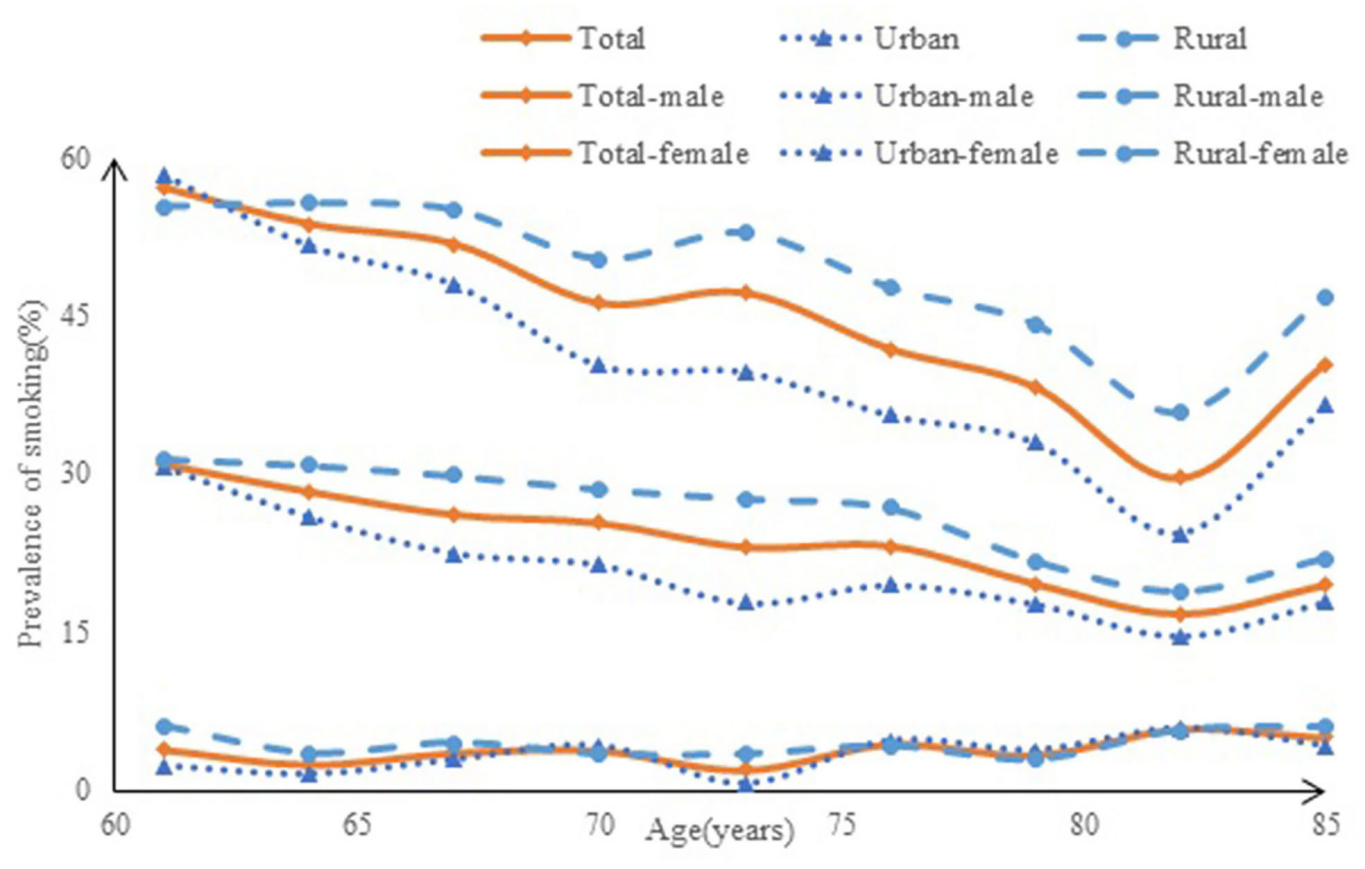
The prevalence of smoking among the elderly in urban and rural areas of China.

**Figure 3:**
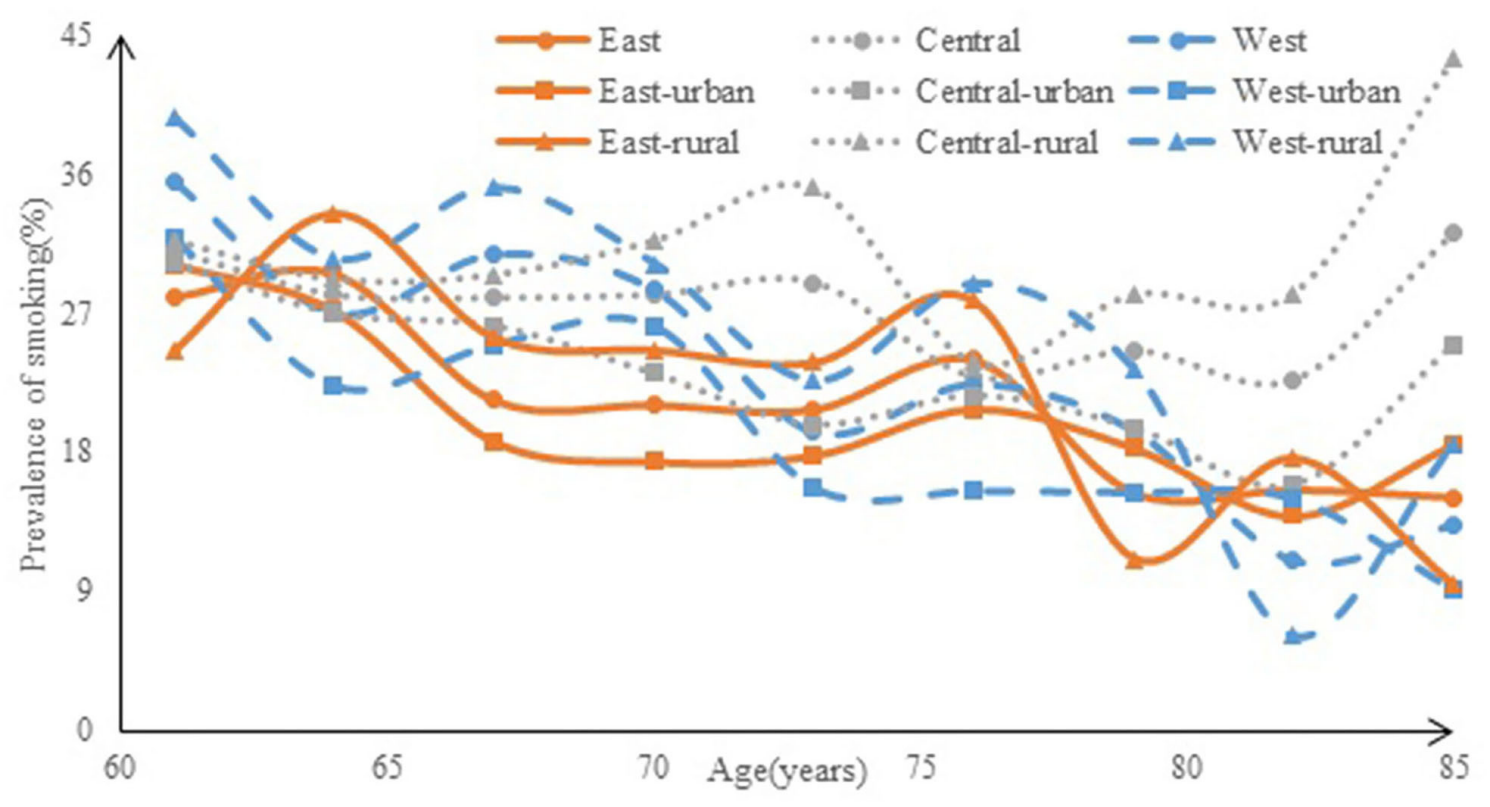
The prevalence of smoking among the elderly by region.

The distribution of smoking prevalence among the elderly by residence and region is shown in Figure 2 (residence) and Figure 3 (region). In total, the rate of smoking among men was 48.9%, and among women was 3.5%; men were more likely to smoke than women (χ^2^ = 1,877.800, *P* < 0.001). In urban areas, the rate of smoking prevalence was 45.6% in men and 2.8% in women, whereas, in the rural areas, it was 52.0% in men and 4.3% in women. According to the Chi-square test, the rural elderly men were more likely to smoke than the urban elderly men (χ^2^ = 13.898, *P* < 0.001), and rural women were also more likely to smoke than urban women (χ^2^ = 5.931, *P* < 0.001). At the regional level, the smoking rate in the eastern region was 23.2%, the central region was 27.7%, and the western region was 26.6%; there was no statistically significant difference in smoking rates between the central and western regions, while the difference between the eastern and western smoking rates and the eastern and central smoking rates were statistically significant, indicating that the smoking situation in the central and western regions was the same, but the smoking situation in the eastern region was better than that in the central and western regions (χ^2^ = 14.381, *P* < 0.001).

### Smoking-Related Knowledge

The response rate for correct answers to all the questions was higher among the urban elderly than the rural elderly (all *P* < 0.001) (Table 2). The question with the highest correct response rate was code 5; 73.9% of the elderly believed that smoking can cause serious diseases. The question with the lowest correct response rate was code 10; 88.8% of the elderly believed that the harm caused by low-tar cigarettes is inconsistent with regular cigarettes.

**Table 2:** Responses to smoking-related knowledge among the urban and rural elderly.

**Code**	**Questions about smoking-related knowledge**	**Yes**	**No (Uncertain)**	**χ^2^**	***P*-value**
		**[*n* (%)]**	**[*n* (%)]**		
1.	Second-hand smoke can cause serious illness among non-smokers.	3,480 (50.0)	3,486 (50.0)	328.220	<0.001
	Urban	2,130 (60.7)	1,377 (39.3)		
	Rural	1,350 (39.0)	2,109 (61.0)		
2.	Second-hand smoke can cause heart disease in adults.	1,976 (28.4)	4,990 (71.6)	176.898	<0.001
	Urban	1,245 (35.5)	2,262 (64.5)		
	Rural	731 (21.1)	2,728 (78.9)		
3.	Second-hand smoke can cause lung diseases in children.	3,071 (44.1)	3,895 (55.9)	346.953	<0.001
	Urban	1,932 (55.1)	1,575 (44.9)		
	Rural	1,139 (32.9)	2,320 (67.1)		
4.	Second-hand smoke can cause lung cancer in adults.	3,136 (45.0)	3,830 (55.0)	377.156	<0.001
	Urban	1,982 (56.5)	1,525 (43.5)		
	Rural	1,154 (33.4)	2,305 (66.6)		
5.	Smoking can cause serious illnesses.	5,145 (73.9)	1,821 (26.1)	64.072	<0.001
	Urban	2,737 (78.0)	779 (22.0)		
	Rural	2,408 (69.6)	1,051 (30.4)		
6.	Smoking can cause stroke (blood clots in the brain that may cause paralysis).	2,389 (34.3)	4,577 (65.7)	50.141	<0.001
	Urban	1,343 (38.3)	2,164 (61.7)		
	Rural	1,046 (30.2)	2,413 (69.8)		
7.	Smoking can cause heart disease.	2,891 (41.5)	4,075 (58.5)	53.603	<0.001
	Urban	1,606 (45.8)	1,901 (54.2)		
	Rural	1,285 (37.1)	2,174 (62.9)		
8.	Smoking can cause lung cancer.	4,700 (67.5)	2,266 (32.5)	159.375	<0.001
	Urban	2,613 (74.5)	894 (25.2)		
	Rural	2,087 (60.3)	1,372 (39.7)		
9.	Smoking can cause erectile dysfunction.	1,104 (15.8)	5,862 (84.2)	42.368	<0.001
	Urban	655 (18.7)	2,852 (81.3)		
	Rural	449 (13.0)	3,010 (87.0)		
10.	Do you think low-tar cigarettes are just as harmful as regular cigarettes?	777 (11.2)	6,189 (88.8)	113.287	<0.001
	Urban	531 (15.1)	2,976 (84.9)		
	Rural	246 (7.1)	3,213 (92.9)		

The average smoking-related knowledge score was 4.12 (SD = 3.068), among which the score for the urban elderly was 4.78 (SD = 3.076) and the rural elderly was 3.44 (SD = 2.907); 55.1% of the elderly had low smoking-related knowledge scores (Table 3). There were more elderly people in rural areas with low smoking knowledge scores, and more elderly people in cities in other segments, the overall score of the urban elderly was better than that of the rural elderly (*P* < 0.001).

**Table 3:** Distribution of smoking-related knowledge scores.

**Scores**	**Urban**	**Rural**	**Overall**	**χ^2^**	***P*-value**
0–4	1,581^a^ (45.1)	2,256^b^ (65.2)	3,837 (55.1)	302.057	<0.001
5–7	1,025^a^ (29.2)	732^b^ (21.2)	1,757 (25.2)		
8–10	901^a^ (25.7)	471^b^ (13.6)	1,372 (19.7)		

a,b *Denote the statistical outcomes of the Chi-square pairwise comparison; the same characteristic indicates significance at P > 0.05, and a different characteristic indicates significance at P < 0.05*.

### Attitudes Toward Smoking

#### Cigarette Packages

**Figure 4:**
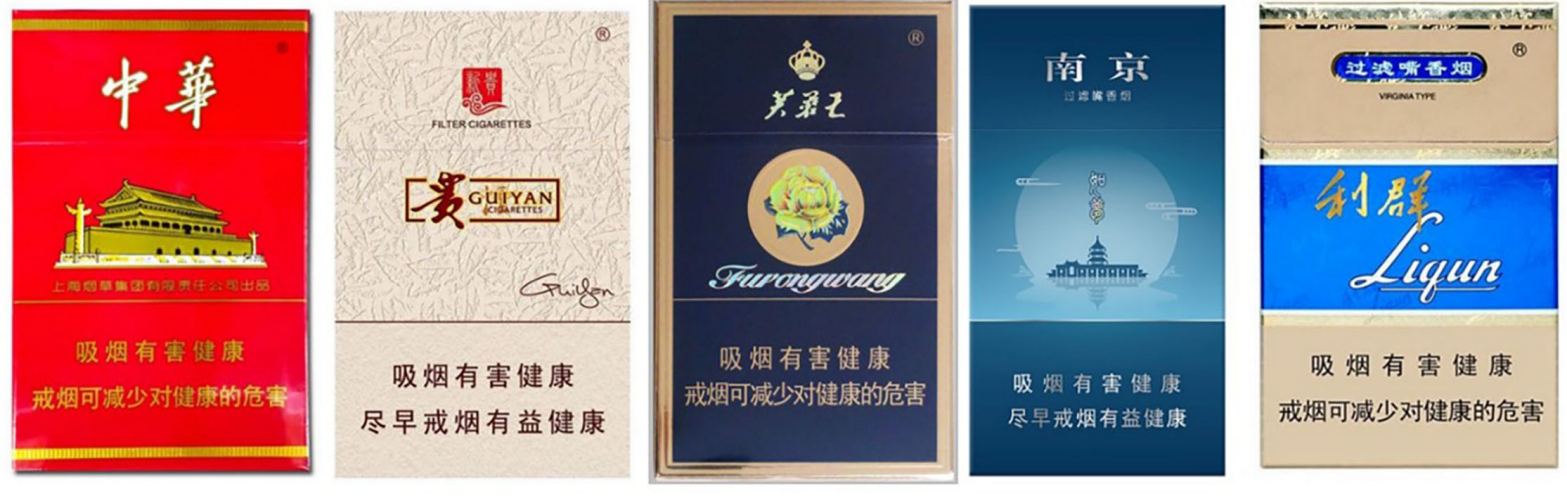
Some common cigarette packages in the Chinese market.

The packaging of Chinese cigarette boxes resembles a delicate artwork; Figure 4 shows some common cigarette packages in the Chinese market. The packet cover has a simple slogan: smoking is harmful to your health, and smoking cessation is healthy as soon as possible. The results of the survey showed that 42.66% of the participants noticed the health warning on cigarette packages, and the urban elderly were more likely to notice it (*P* < 0.001); people who smoked were more likely to notice the warning in both urban (*P* < 0.001) and rural (*P* < 0.001) areas than those who did not smoke (Table 4). Moreover, only 29.91% of the participants reported that the health warning on the packets made them think about quitting; most people (67.82%) thought the warning was useless, and there was no significant difference in this understanding between smokers and non-smokers in both urban (*P* = 0.387) and rural (*P* = 0.050) areas.

**Table 4:** Responses to the health warning on cigarette packages among urban and rural elderly.

**Attitudes toward smoking**	**Urban**	**Rural**	**χ^2^**	***P*-value**	**Urban**				**Rural**			
	**[*n* (%)]**	**[*n* (%)]**										
					**Smoker**	**Non-smoker**	**χ^2^**	***P*-value**	**Smoker**	**Non-smoker**	**χ^2^**	***P*-value**
					**[*n* (%)]**	**[*n* (%)]**			**[*n* (%)]**	**[*n* (%)]**		
Have you noticed any health warning on cigarette packages?			59.913	<0.001			616.242	<0.001			496.905	<0.001
Yes	1,591^a^ (45.4)	1,381^b^ (39.9)			669^a^ (83.1)	922^b^ (34.1)			669^a^ (68.3)	712^b^ (28.7)		
No	1,050^a^ (29.9)	1,340^b^ (38.7)			111^a^ (13.8)	939^b^ (34.8)			260^a^ (26.6)	1,080^b^ (45.3)		
Didn't see any cigarette packages	866^a^ (24.7)	738^b^ (21.3)			25^a^ (3.1)	841^b^ (31.1)			50^a^ (5.1)	688^b^ (27.7)		
Have warning labels on cigarette packages led you to think about quitting? (Sample size:1,364)			22.911	0.001			1.900	0.387			5.999	0.050
Yes	163^a^ (24.0)	245^b^ (35.8)			153^a^ (23.8)	10^a^ (27.0)			233^a^ (36.1)	12^a^ (30.8)		
No	501^a^ (73.7)	424^b^ (62.0)			476^a^ (74.0)	25^a^ (67.6)			400^a^ (62.0)	24^a^ (61.5)		
Don't know	16^a^ (2.4)	15^a^ (2.2)			14^a^ (2.2)	2^a^ (5.4)			12^a^ (1.9)	3^b^ (7.7)		
Randomly show 1 of 5 pictures to respondents (Figure 5)			3.846	0.427			0.886	0.927			5.188	0.269
1. Smoking and second-hand smoking cause lung cancer.	607 (18.7)	540 (20.3)			136 (18.3)	471 (18.8)			150 (19.9)	390 (20.4)		
2. Smoking causes chronic obstructive pneumonia.	675 (20.8)	519 (19.5)			156 (21.0)	519 (20.7)			165 (21.9)	354 (18.5)		
3. Smoking causes yellow teeth, bad breath, and periodontal disease.	656 (20.2)	551 (20.7)			144 (19.4)	512 (20.4)			151 (20.1)	400 (20.9)		
4. Smoking may cause impotence.	666 (20.5)	523 (19.6)			151 (20.4)	515 (20.5)			149 (19.8)	374 (19.5)		
5. Smoking causes peripheral vascular disease.	646 (19.9)	533 (20.0)			155 (20.9)	491 (19.6)			137 (18.2)	396 (20.7)		
If you see such a health warning on a cigarette package, would you consider to stop smoking? (Sample size:1,780)			0.234	0.890			0.313	0.855			1.326	0.515
Yes	791^a^ (47.2)	48^a^ (46.6)			303^a^ (40.2)	18^a^ (37.5)			488^a^ (52.9)	30^a^ (54.5)		
No	746^a^ (44.5)	45^a^ (43.7)			378^a^ (50.1)	26^a^ (54.2)			368^a^ (39.9)	19^a^ (34.5)		
Don't know/Refuse to answer	140^a^ (8.3)	10^a^ (9.7)			73^a^ (9.7)	4^a^ (8.3)			67^a^ (7.3)	6^a^ (10.9)		
Do you support printing such a pictorial warning (Figure 5) on cigarette packages?			9.999	0.007			64.628	<0.001			19.212	<0.001
Yes	2,201^a^ (62.8)	2,270^b^ (65.6)			452^a^ (56.1)	1,749^b^ (64.7)			666^a^ (68.0)	1,604^a^ (64.7)		
No	711^a^ (20.3)	601^b^ (17.4)			243^a^ (30.2)	468^b^ (17.3)			189^a^ (19.3)	412^a^ (16.6)		
Don't know	595^a^ (17.0)	588^a^ (17.0)			110^a^ (13.7)	485^b^ (17.9)			124^a^ (12.7)	464^b^ (18.7)		

a,b *Are the results of the Chi-square test pairwise comparison; the same characteristic indicates significance at P > 0.05, and a different characteristic indicates significance at P < 0.05*.

**Figure 5:**
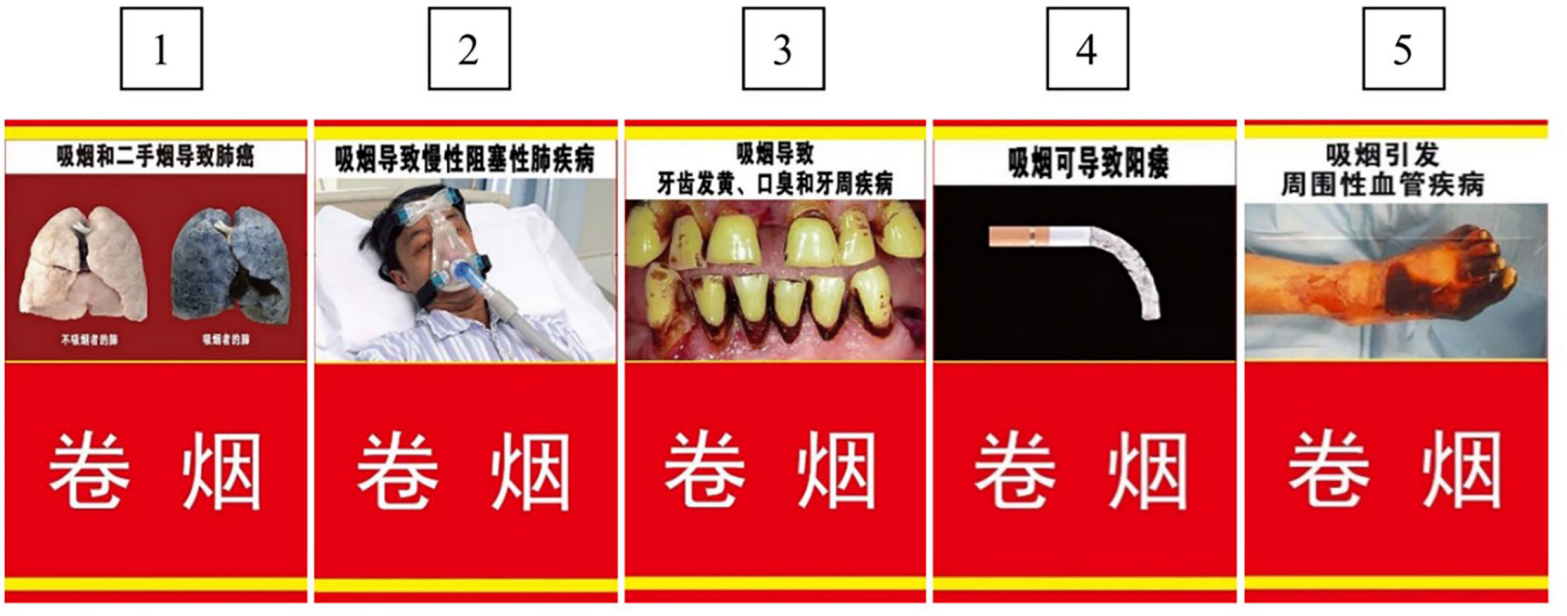
Five pictures showing adverse effects of smoking.

When these pictures were replaced with pictures that affect human health such as those in Figure 5, 47.13% of the participants said that the warning led them to think about quitting; the rate of support for the pictures increased to 17.22%, and there was no significant difference in this understanding between smokers and non-smokers in urban (*P* = 0.855) or rural (*P* = 0.515) areas. Thus, the pictures in Figure 5 were more effective than those in Figure 4 to make participants willing to quit smoking (Table 5), because the harmful effects of smoking are more obvious in Figure 5 (*P* < 0.001). In total, 64.18% of the participants supported printing such a pictorial warning (Figure 5) on cigarette packages, and the rural elderly were more supportive of it (*P* = 0.007); non-smokers were more supportive of printing these pictures than smokers in both urban (*P* < 0.001) and rural (*P* < 0.001) areas.

**Table 5:** Comparison of two types of pictures on cigarette packets to stop smoking.

**Stop smoking**	**Figure 4**	**Figure 5**	**χ^2^**	***P*-value**
Yes	408^a^ (29.9)	839^b^ (47.1)	185.879	<0.001
No	925^a^ (67.8)	791^b^ (44.4)		
Don't know/Refuse	31^a^ (2.3)	150^b^ (8.4)		

a,b *Are the statistical outcomes of the Chi-square test pairwise comparison; the same characteristic shows significance at P > 0.05, and a different characteristic shows significance at P < 0.05*.

In summary, cigarette packets still play an important role in tobacco control, and printing vivid pictures of adverse health consequences on cigarette packs can help reduce smoking prevalence among the elderly.

### Tobacco Taxation

Only 36.52% of the participants supported increasing the tax on cigarettes and retail price of cigarettes; the urban elderly were more supportive of this (*P* < 0.001), and non-smokers were more supportive of it than smokers in both urban (*P* < 0.001) and rural (*P* < 0.001) areas (Table 6).

**Table 6:** Responses to tobacco taxation among the urban and rural elderly in China.

**Questions about increasing taxation and retail price of cigarettes**	**Urban** **[*n* (%)]**	**Rural** **[*n* (%)]**	**χ^2^**	***P*-value**	**Urban**				**Rural**			
					**Smoker**	**Non-smoker**	**χ^2^**	***P*-value**	**Smoker**	**Non-smoker**	**χ^2^**	***P-*value**
					**[*n* (%)]**	**[*n* (%)]**			**[*n* (%)]**	**[*n* (%)]**		
Do you support an increase in the taxation and retail price of cigarettes?			66.015	<0.001			367.060	<0.001			160.767	<0.001
Yes	1,444^a^ (41.2)	1,100^b^ (31.8)			205^a^ (25.5)	1,239^b^ (45.9)			255^a^ (26.0)	845^b^ (34.1)		
No	914^a^ (26.1)	1,041^b^ (30.1)			419^a^ (52.0)	495^b^ (18.3)			448^a^ (45.8)	593^b^ (23.9)		
Don't know/No opinion/Refuse	1,149^a^ (32.8)	1,318^b^ (38.1)			181^a^ (22.5)	968^b^ (35.8)			276^a^ (28.2)	1,042^b^ (42.0)		
If there was an increase in the tax on cigarettes, do you think part of the money should be spent on tobacco control (e.g., support cessation services and warning against tobacco hazards advocacy)?			18.595	<0.001			76.204	<0.001			28.419	<0.001
Yes	2,351^a^ (67.0)	2,148^b^ (62.1)			486^a^ (60.4)	1,865^b^ (69.0)			578^a^ (59.0)	1,570^b^ (63.3)		
No	242^a^ (6.9)	278^a^ (8.0)			110^a^ (13.7)	132^b^ (4.9)			117^a^ (12.0)	161^b^ (6.5)		
Don't know/No opinion/Refuse	914^a^ (26.1)	1,033^b^ (29.9)			209^a^ (26.0)	705^a^ (26.1)			284^a^ (29.0)	749^a^ (30.2)		
If there was an increase in the tax on cigarettes, do you think part of the money should be spent on paying some of the costs of health insurance?			31.319	<0.001			33.284	<0.001			23.424	<0.001
Yes	2,759^a^ (78.7)	2,545^b^ (73.6)			600^a^ (74.5)	2,159^b^ (79.9)			701^a^ (71.6)	1,844^a^ (74.4)		
No	153^a^ (4.4)	143^a^ (4.1)			64^a^ (8.0)	89^b^ (3.3)			66^a^ (6.7)	77^b^ (3.1)		
Don't know/No Opinion/Refuse	595^a^ (17.0)	771^b^ (22.3)			141^a^ (17.5)	454^a^ (16.8)			212^a^ (21.7)	559^a^ (22.5)		

a,b *Are the statistical outcomes of Chi-square test pairwise comparison; the same characteristic indicates significance at P > 0.05, and a different characteristic indicates significance at P < 0.05*.

If there was an increase in the tax on cigarettes, 64.59% of the participants supported that part of the money should be spent on tobacco control; here too, the urban elderly showed more support (*P* < 0.001). Further, 76.14% of the participants supported the notion that part of the money should be spent on paying the costs of health insurance, and the urban elderly people were more supportive of this (*P* < 0.001). Regarding these two issues, non-smokers expressed greater support than smokers, in both urban and rural areas (all *P* < 0.001).

In China, a small number of elderly people supported an increase in tobacco taxation, and the supporters preferred to spend this part of the money on health insurance.

### Sociodemographic Factors Associated With Smoking Status

A stepwise multiple logistic regression model was used to explore the factors that influenced smoking behavior among the elderly. Sociodemographic variables and smoking-related knowledge scores with statistically significant differences were used as independent variable X, and smoking status was set as dependent variable Y (0 = no smoking, 1 = smoking); the results of the model results are shown in Table 7. Gender, age, region, education, knowing about e-cigarettes, rules about smoking at home, and scores of smoking-related knowledge were major risk factors for smoking among the elderly; participants who were male, had a lower age (60–69 years), belonged to the central region, had an elementary education level or below, were allowed or never allowed or had no rules about smoking at home, had knowledge about e-cigarettes, and had low (0–4) or high (8–10) smoking-related knowledge scores were more likely to smoke. However, region was not a major factor for smoking among the urban elderly, and rules about smoking at home were not a major factor among the rural elderly.

**Table 7:** Results of the logistic regression for sociodemographic characteristics associated with smoking status.

**Variable**	**Overall**		**Urban**		**Rural**	
	**OR**	**95%CI**	**OR**	**95%CI**	**OR**	**95%CI**
**Gender**						
Female	1.00		1.00		1.00	
Male	26.234^**^	(21.528, 31.969)	31.260^**^	(22.942, 42.594)	23.889^**^	(18.431, 30.962)
**Age**						
Low (60–69 years)	1.00		1.00		1.00	
Middle (70–79 years)	0.501^**^	(0.393, 0.640)	0.422^**^	(0.295, 0.604)	0.556^**^	(0.400, 0.775)
High (80 years or above)	0.688^**^	(0.533, 0.887)	0.756	(0.523, 1.120)	0.626^**^	(0.443, 0.886)
**Region**						
East	1.00		-		1.00	
Central	1.265^**^	(1.067, 1.499)	-	-	1.530^**^	(1.209, 1.937)
West	1.026	(0.865, 1.217)	-	-	1.094	(0.875, 1.367)
**Education level**						
Elementary education and below	1.00		1.00		1.00	
Secondary education	0.475^**^	(0.323, 0.698)	0.493^**^	(0.323, 0.752)	0.107^*^	(0.013, 0.908)
Higher education and above	0.483^**^	(0.332, 0.701)	0.491^**^	(0.331, 0.728)	0.117^*^	(0.014, 0.991)
**Have you ever heard of e-cigarettes?**						
No	1.00		1.00		1.00	
Yes	1.764^**^	(1.486, 2.095)	1.686^**^	(1.334, 2.123)	1.844^**^	(1.419, 2.395)
**Rules about smoking at home**						
Allowed	1.00		1.00		-	
Not allowed, but with exceptions	0.308^**^	(0.172, 0.552)	0.117^**^	(0.038, 0.358)	-	-
Never allowed	0.981	(0.538, 1.790)	0.429	(0.138, 1.329)	-	-
No rules	1.179	(0.684, 2.147)	0.628	(0.202, 1.951)	-	-
Don't know/Refuse	0.640	(0.355, 1.153)	0.415	(0.134, 1.288)	-	-
**Scores of smoking-related knowledge**						
0–4	1.00		1.00		1.00	
5–7	0.674^**^	(0.558, 0.814)	0.606^**^	(0.465, 0.791)	0.751^*^	(0.570, 0.989)
8–10	1.023	(0.834, 1.256)	0.946	(0.717, 1.247)	1.136	(0.831, 1.553)

* *Significant at P < 0.05*;

** *Significant at P < 0.01*.

Interestingly, when smoking was not allowed at home but there were exceptions to the rule, people found it easier not to smoke, whereas, those with low or high smoking-related knowledge scores were more likely to smoke.

### Decomposition Analysis

This study aimed to determine the disparities in smoking prevalence and factors associated with it between the rural and urban elderly in China. The disparities between the two groups are shown in Table 8. In total, 53.45% of the disparities were enlightened by the factors considered, and 46.55% were caused by the factor of urban and rural residence. In addition, the results confirmed that gender (-1.62%), age (-2.03%), region (13.68%), knowing about e-cigarettes (5.17%), rules about smoking at home (3.95%), and smoking-related knowledge scores (42.85%) significantly explained the variations in smoking behavior (*P* < 0.05).

**Table 8:** The Fairlie decomposition model of smoking status between urban and rural.

**Terms of decomposition**	**Smoking status**			
Difference	0.05348886			
Explained (%)	0.02858828 (53.45)			
Non-explained (%)	0.02490058 (46.55)			
**Contribution to differences**	**β**	***P*** **-value**	**Contribution (%)**	**[95%CI]**
**Explained**				
Gender	0.0132741	<0.001	−1.62	(0.0119426, 0.0146057)
Age	−0.0005792	0.008	−2.03	(−0.0010063, −0.0001521)
Residential status	−0.0004633	0.606	46.43	(−0.002223, 0.0012964)
Region	0.0039109	<0.001	13.68	(0.0021276, 0.0056941)
Educational level	0.0101457	0.054	35.49	(−0.0001549, 0.0204464)
Employment status	0.0014767	0.842	7.57	(−0.0130856, 0.0160389)
Annual income	0.0021653	0.661	−51.45	(−0.0075007, 0.0118312)
Have you ever heard of e-cigarettes?	−0.0147092	<0.001	5.17	(−0.0212032, −0.0082152)
Rules about smoking at home	0.0011291	0.001	3.95	(0.0004849, 0.0017732)
Scores of smoking-related knowledges	0.0122492	<0.001	42.85	(0.0063375, 0.0181609)

## Discussion

To the best of our knowledge, this is the first large-scale comparative study to conduct a decomposition analysis of the prevalence of smoking between rural and urban areas, specifically focusing on the elderly population (aged 60 years and above) in China. This study revealed the distribution of smoking prevalence by gender and region, providing new empirical evidence for smoking behavior among the elderly. In addition, it assessed smoking-related knowledge, attitudes, and demographic characteristics of the elderly in urban and rural areas in China and analyzed the decomposition of each factor.

These results showed an overall 25.6% prevalence rate for smoking among the elderly, where the prevalence in urban areas was 23.0% and in rural areas was 28.3%. In total, the rate of prevalence was 48.9% in men and 3.5% in women; in rural areas, the rate was 45.6% in men and 2.8% in women, whereas, in urban areas, it was 52.0% in men and 4.3% in women. However, in both urban and rural China, smoking prevalence among men was much higher than that among women (overall: OR = 26.234; urban: OR = 31.260; rural: OR = 23.889). At the regional level, the rate of smoking prevalence in eastern, Central, and western China was 23.2%, 27.7%, and 26.6%, respectively; people in the central region were more likely to smoke than those in the eastern and western regions (central region, OR=1.265), but this difference was only found in rural areas (central region, OR = 1.530).

According to the *China Smoking Hazardous Health Report 2020*, released by the CCDC in 2021, the smoking prevalence among people aged 15 and above was 26.6% in 2018; the smoking prevalence in men was 50.5% and in women was 2.1% (21). Moreover, the prevalence in urban and rural areas was 25.1 and 28.9%, respectively (21). Overall, our results are similar to those of Ding et al. (22); smoking rates decreased with age in both urban and rural areas, and in both men and women, the smoking rate among the elderly was slightly lower than the national average in China. Notably, in both urban and rural areas, the smoking rate among men decreased with age, but hardly changed with age among women.

Our study revealed that the rate of correct responses to all smoking-related problems was significantly higher among the urban elderly compared with the rural elderly, perhaps because urban residents have better educational opportunities and can learn more about the effects of smoking (20). The stepwise multiple logistic regression model showed that gender, age, region, education, knowing about e-cigarettes, rules about smoking at home, and smoking-related knowledge scores were major risk factors for smoking in the elderly; age and education were found to be protective factors against smoking. However, it was not that people with higher smoking-related knowledge scores are less likely to smoke; only moderate smoking-related knowledge scores served as protective factors against smoking. Some studies have shown that this may be because people with higher scores are those who had better academic qualifications, occupations that are more inclined to mental work or retired, may be subject to greater mental stress or bored, and compared with elderly people who scored low, those with high scores were often from urban areas, with a higher constant pension, and thus, had stronger purchasing power for cigarettes (6, 7, 20).

In this study, we found that living alone, annual income, and occupation are no longer the main factors influencing smoking behavior among the elderly smoking; these results are different from previous studies (23, 24). It is worth noting that people who had heard of e-cigarettes were more likely to smoke; however, this may be because smokers are more likely to hear about e-cigarettes, which is consistent with existing survey results among adults (25–27). At the same time, we found that family rules can also play a role, especially where smoking at home is not allowed but there are exceptions to the rule (overall: OR = 0.308; urban: OR = 0.117); nevertheless, this factor was only significant in urban families.

Elderly people in both urban and rural areas supported printing photos of the serious health hazards of smoking on the cover of cigarette packs. At present, most of the designs on Chinese cigarette packs are artistic and exquisite, which elicits a desire for collection and is not conducive to smoking control (28). An appeal should be urgently made to the Chinese government to draw attention to this detail; the existing cover designs should be changed and replaced with pictures showing the harmful effects of smoking (29, 30).

In China, a small number of elderly people supported an increase in tobacco taxation, and the supporters preferred to spend this money to cover health insurance costs. Studies by scholars such as Nigar Nargis and Xiao Hu showed that the current price of cigarettes in China is decreasing, and people who buy low-priced cigarettes often smoke more frequently. The scholars believe that raising the price of cigarettes and thus making cigarettes less affordable could have a larger impact on reducing the number of cigarettes smoked, particularly for elderly smokers, smokers with low levels of education, and smokers from rural areas, which, in turn, could benefit these vulnerable groups by reducing their health burden and economic costs attributed to smoking (31, 32).

Using the Fairlie model, we conducted a decomposition analysis and found that gender (-1.62%), age (-2.03%), region (13.68%), knowing about e-cigarettes (5.17%), rules about smoking at home (3.95%), and scores of smoking-related knowledges (42.85%) were associated with rural-urban disparities in smoking.

Based on the differences in smoking between the urban and rural elderly population, our conclusions have strong policy recommendations. First, we need to focus on the men living in rural areas, and the elderly with low levels of education; Chinese family doctors should assume the function of health education to help them realize the health hazards of smoking. Second, based on regional disparities, we need to pay attention to the elderly smoking situation in the central and western regions of China; it has been recommended to funds for tobacco control should be allotted to the central and western regions (20). Finally, we suggest that the Chinese government should increase the public awareness of the health hazards of smoking, reduce or ban the advertising of e-cigarettes (25), compel pictures of the health hazards of tobacco to be printed on the cover of cigarette packets (29, 30), and increase the tax on tobacco and use the money to cover health insurance, thus benefiting public health and welfare (31–33). In summary, smoking is still highly prevalent among the elderly in China, and the government should continue to increase tobacco control efforts to promote healthy lifestyles for the elderly.

### Limitations

This study has several limitations which should be noted. First, China is a country with a very large elderly population; the amount of cross-sectional survey data used in this study was limited and did not cover the entire elderly population. Second, smoking among the elderly is affected by many factors, and only some of these were analyzed in the current study. Despite these limitations, the results are useful in comparing the differences in smoking between the elderly in urban and rural areas in China, and provide guidelines for strategies to control smoking among the elderly in China. We aim to collect more data and analyse more factors in follow-up studies to verify the rationality of our results.

## Conclusion

This study focused on the differences in smoking between the elderly residents of urban and rural areas in China. Smoking among the urban elderly was significantly less prevalent than among the rural population. Factors including education, region, and propaganda need to be addressed to reduce the gap between urban and rural health hazards in China. Our findings can help provide empirical evidence to develop health policies and strategies to reduce the differences in health risk factors between China's urban and rural elderly population.

## Data Availability Statement

The datasets analyzed in this study can be found on the GATS website: http://ghdx.healthdata.org/record/china-global-adult-tobacco-survey-2018 (accessed on 26 January 2022).

## Ethics Statement

The data for this study were taken from the GATS survey, which is organized by the CCDC, and has been approved by the National Ethics Committee, the data analyzed here are available in the public domain. Therefore, separate ethical approval was not required for this study.

## Author Contributions

LY and JS designed the study. ZZ, YX, and JW controlled the quality of the data and performed statistical analysis. LY, MD, and LL managed and checked all the data. LY, ZZ, JW, and YX contributed to manuscript preparation, editing, and review. All authors have read, checked, and approved the final manuscript.

## Conflict of Interest

The authors declare that the research was conducted in the absence of any commercial or financial relationships that could be construed as a potential conflict of interest.

## Publisher's Note

All claims expressed in this article are solely those of the authors and do not necessarily represent those of their affiliated organizations, or those of the publisher, the editors and the reviewers. Any product that may be evaluated in this article, or claim that may be made by its manufacturer, is not guaranteed or endorsed by the publisher.

## Abbreviations

CCDC: Chinese Center for Disease Control and Prevention
GATS: Global Adult Tobacco Survey
OR: Odds ratios.
